# Development of cell differentiation in the transition to multicellularity: a dynamical modeling approach

**DOI:** 10.3389/fmicb.2015.00603

**Published:** 2015-06-23

**Authors:** Emilio Mora Van Cauwelaert, Juan A. Arias Del Angel, Mariana Benítez, Eugenio M. Azpeitia

**Affiliations:** ^1^Laboratorio Nacional de Ciencias de la Sostenibilidad, Instituto de Ecología, Universidad Nacional Autónoma de MéxicoMexico, Mexico; ^2^Centro de Ciencias de la Complejidad, Universidad Nacional Autónoma de MéxicoMexico, Mexico; ^3^Programa de Doctorado en Ciencias Biomédicas, Universidad Nacional Autónoma de MéxicoMexico, Mexico; ^4^Institut National de Recherche en Informatique et en Automatique Project-Team Virtual Plants joint with CIRAD and INRAMontpellier, France; ^5^Departamento de Ecología Funcional, Instituto de Ecología, Universidad Nacional Autónoma de MéxicoMexico, Mexico

**Keywords:** multicellularity, differentiation, multiscale modeling, dynamical patterning modules, cooperation, defection

## Abstract

Multicellularity has emerged and continues to emerge in a variety of lineages and under diverse environmental conditions. In order to attain individuality and integration, multicellular organisms must exhibit spatial cell differentiation, which in turn allows cell aggregates to robustly generate traits and behaviors at the multicellular level. Nevertheless, the mechanisms that may lead to the development of cellular differentiation and patterning in emerging multicellular organisms remain unclear. We briefly review two conceptual frameworks that have addressed this issue: the cooperation-defection framework and the dynamical patterning modules (DPMs) framework. Then, situating ourselves in the DPM formalism first put forward by S. A. Newman and collaborators, we state a hypothesis for cell differentiation and arrangement in cellular masses of emerging multicellular organisms. Our hypothesis is based on the role of the generic cell-to-cell communication and adhesion patterning mechanisms, which are two fundamental mechanisms for the evolution of multicellularity, and whose molecules seem to be well-conserved in extant multicellular organisms and their unicellular relatives. We review some fundamental ideas underlying this hypothesis and contrast them with empirical and theoretical evidence currently available. Next, we use a mathematical model to illustrate how the mechanisms and assumptions considered in the hypothesis we postulate may render stereotypical arrangements of differentiated cells in an emerging cellular aggregate and may contribute to the variation and recreation of multicellular phenotypes. Finally, we discuss the potential implications of our approach and compare them to those entailed by the cooperation-defection framework in the study of cell differentiation in the transition to multicellularity.

## 1. Introduction

The evolution of multicellular organisms with some degree of cell differentiation and characteristic spatial arrangements has been identified as one of the major transitions in the evolutionary history of life (Maynard-Smith and Szathmary, 2000). Indeed, with the formation of many-celled organisms came a qualitative change in scale and the specialization of coexisting cell types; cell differentiation became spatial and not only temporal (Michod, 2007; Mikhailov et al., 2009).

Multicellular organisms, here defined broadly as an integrated mass of cells with spatial differentiation have evolved at least 25 times (Grosberg and Strathmann, 2007). Nevertheless, the mechanisms that lead or have led to the formation of stable patterns of cell differentiation in emerging multicellular organisms are not yet clear (Maynard-Smith and Szathmary, 2000). There are two main frameworks dealing with the process of cellular differentiation and patterning in emerging multicellular organisms. Here we refer to them as the cooperation-defection framework (Michod and Roze, 2001; Michod and Herron, 2006) and the dynamical patterning modules (DPMs) framework (Newman et al., 2006; Newman and Bhat, 2009). The cooperation-defection framework focuses on the conditions that allow and could produce cell differentiation and patterning in a group of cells that behave as cooperators or defectors (cells that contribute or not to the group fitness, respectively) in emerging multicellular organisms. On the other hand, the DPMs framework focuses on the mechanisms involved in cell differentiation and patterning through cell-to-cell interactions and physicochemical processes. Although both approaches deal with similar questions and can lead to complementary explanations, in some cases they can also imply contrasting assumptions and ideas.

In this article we first provide a description of both frameworks in the context of the transition to multicellularity. Second, we elaborate on the potential role of two specific DPMs in cell differentiation, and present a working hypothesis for the development of cell differentiation in the transition to multicellularity. Third, we present a mathematical model that illustrates this proposal and can be used to perform *in silico* tests. Finally, in light of our results and other available data, we discuss the scope, limitations and predictions of our proposal and its possible impact on the cooperation-defection framework.

## 2. Two frameworks for studying the origin of cell differentiation and patterning in the transition to multicellularity

### 2.1. The cooperation–defection framework

In the cooperation-defection framework (as understood in the context of game theory) it is considered that cell differentiation involves differences in fitness among components of the multicellular organism. For instance, germinal cells, which divide and reproduce, would have a higher fitness at the individual level in comparison to somatic cells, which do not reproduce and instead contribute to the group fitness (Michod and Roze, 2001). Hence, under this framework germinal cells are thought of as defector cells and somatic cells as cooperative cells (Michod and Roze, 2001). Multicellular organisms with different cellular fitnesses at the individual level might always be affected by defector cells that use the resources of cooperative individuals for their own benefit without contributing anything in return (Nowak, 2006), therefore destabilizing the entire organism (Michod and Roze, 2001).

This raises two important questions: (1) how can cooperative behaviors and thus cell differentiation be robustly maintained in emerging multicellular organisms? and (2) how does a cell attain a cooperating or a defecting behavior? The first question is mainly an evolutionary matter, and considerable effort has been invested in answering it. It has been suggested that cell differentiation and patterning cannot appear without mechanisms that enhance and maintain cooperative behaviors in the face of defector cells. For example, when some conditions are met, such as a given spatial structure (Ohtsuki et al., 2006), high relatedness among the individuals of the group (Grosberg and Strathmann, 2007) or the presence of conflict-mediation mechanisms (Travisano and Velicer, 2004), cooperative behaviors can evolve and become fixed in populations, leading to the maintenance of cell differentiation (Michod, 2003; Gilbert et al., 2007; Nowak et al., 2010; Powers et al., 2011). Michod (2007) suggests that once a mass of undifferentiated cells reaches a threshold size, division of labor becomes beneficial for the group even if it implies that some of the cell types will have a relatively low fitness, leading to or maintaining cell differentiation. This is followed by the transformation of the individual cells into essential components of the group fitness, and finally by their spatial organization (Michod, 2007).

To answer the second question, it has been proposed that cooperating and defecting behaviors are intrinsic to individual cells and that genetic differences or specific genotypes could underlie these two types of behavior (Kirk et al., 1987; Michod and Roze, 2001; Travisano and Velicer, 2004; Thompson et al., 2013). It is of course possible that genetic differences could explain changes in cell behavior, but it would be desirable to aim at explanations that account for cellular differentiation and patterning in sets of cells that, much like in most multicellular organisms, do not exhibit critical differences in their genotypes. On the other hand, differences between cell behavior have also been attributed to changes in gene activation profiles within an organism. In fact, in some organisms, differentiation and cell patterning are partially affected by the differential expression of certain genes, such as *regA* in the development of *Volvox carterii* (Michod, 2007). Nevertheless, it is not clear how *regA* or other genes related to cooperation are regulated in a position- and time-dependent manner (Maynard-Smith and Szathmary, 2000), and further investigation of the developmental processes that produce spatial patterns of gene expression is needed (Bonner, 2001).

### 2.2. The dynamical patterning module framework

DPMs are sets of well-conserved molecules in interaction with generic physical processes (i.e., those processes common to living and nonliving chemically and mechanically excitable systems). The DPMs framework postulates that DPMs couple cells and give rise to steady differences among them, as well as to their spatial arrangement. This framework proposes that some molecules already present in single cells may mobilize physicochemical processes that, at the multicellular scale, yield the organization and patterning of multicellular masses. In fact, some authors suggest that the variety of reproducible, yet plastic, patterns generated by DPMs could have been of particular importance in the emergence of specific arrangements at the origin of multicellularity (Newman and Bhat, 2008; Niklas, 2014). Preliminary sets of basic DPMs have been postulated for animal and plant development (Newman and Bhat, 2008, 2009; Hernández-Hernández et al., 2012; Newman, 2012). For example, in both lineages a DPM for position-dependent differentiation has been identified in the interaction between generic patterning mechanisms (e.g., reaction-diffusion processes) and relatively well-conserved molecules that may move among cells (Newman and Bhat, 2009; Hernández-Hernández et al., 2012).

In turn, the physicochemical processes mobilized by these DPMs should be able to interact with intracellular and multistable biochemical networks leading to stable patterns of differentiated cells (Hogeweg, 2000; Ten-Tusscher and Scheres, 2011). The importance of coupling among multistable cells as a patterning mechanism has also been illustrated by diverse modeling approaches (Furusawa and Kaneko, 2002; Benítez et al., 2008; Azpeitia et al., 2010; Salazar-Ciudad, 2010; Inoue and Kaneko, 2013).

The problem of cell differentiation and patterning evolution has been recognized by both the cooperation-defection and DPMs frameworks, each with very different assumptions. Here, we explore the application of the DPMs framework to the origin of cell differentiation and patterning in the transition to multicellularity.

## 3. DPMs framework for the development of patterns of differentiated cells in the transition to multicellularity

It has been argued that some of the key ingredients for the transition to multicellularity are cell adhesion and cell-to-cell communication (Grosberg and Strathmann, 2007; Rokas, 2008b; Niklas, 2014). Therefore, in this work, we postulate that the fundamental DPMs for the appearance of multicellular organisms are those involved in communication and adhesion. However, for these DPMs to be involved in the evolution of cell differentiation and patterning during the emergence of multicellular organisms, they (i) should be capable of generating cell patterning among multistable cells and (ii) the molecules involved in these DPMs should precede or evolve during the transition to multicellularity. In the following sections we elaborate on these two matters and then postulate our main hypothesis.

### 3.1. Adhesion and communication DPMs are capable of generating cell patterning among multistable cells

It has been shown that DPMs are capable of generating patterns by coupling cells' properties (e.g., Furusawa and Kaneko, 2002; Newman and Bhat, 2008, 2009; Zhu et al., 2010a). For example, some authors have pointed out that mechanical forces exerted on an extracellular matrix can couple cells in a tissue and regulate cellular differentiation and behavior (Ingber, 2002). This is also the case for the adhesion and the communication DPMs. It has been experimentally and theoretically demonstrated that cells with different adhesive properties can generate organized cell patterns (Armstrong and Armstrong, 1984; Swat et al., 2012), and that communication-related DPMs like those involving diffusion and reaction of molecules play an important role in coupling cells and producing patterns at the multicellular or tissue scale. Some of these mechanisms were explored by Turing (1952) with the reaction-diffusion model and by Meinhardt and Gierer (1974) with the lateral-inhibition model. In addition, there is now vast evidence confirming the existence and relevance of these mechanisms in living organisms (e.g., Sternberg, 1988; Affolter and Basler, 2007; Kondo and Miura, 2010; Lander, 2011; Rogers and Schier, 2011). For instance the lateral inhibition and the diffusion DPMs can couple neighboring cells and have proven to be critical for the generation of gradients and patterns of molecules involved in the development of plant, animal and microbial systems. These DPMs have been found to render periodic arrangements in leaves, animal skin and developing bones, and cellular filaments (Newman and Bhat, 2008, 2009; Zhu et al., 2010a,b; Benítez et al., 2011; Hernández-Hernández et al., 2012; Watanabe and Kondo, 2015).

DPMs generate dynamic patterns that provide position-dependent information during development. Moreover, cells in developing organisms have multistable biochemical networks whose steady states have been interpreted as specific cell types (Kauffman, 1969; Thomas, 1973). Thus, position-dependent information might affect the dynamics of intracellular biochemical networks and bias the cells to a particular differentiated state. This interaction between multistable networks and DPMs has been illustrated by modeling in diverse study systems. For example, work in *Arabidopsis thaliana* shows that the spatial organization of the cellular types identified in the root stem cell niche emerges from the coupling of multistable gene networks via molecules that can move among neighboring cells (Azpeitia et al., 2010). Similarly, a model employed to explain the segmentation process in *Drosophila melanogaster* shows the importance and generality of the coupling of multistable networks for pattern formation during development (von Dassow and Odell, 2002; Jaeger and Reinitz, 2006). In another theoretical work, Furusawa and Kaneko (2002) showed that when an aggregate of virtual multistable and coupled cells grows, new steady states (virtual cell types) arise as a consequence of the emergence of microenvironments inside the aggregate.

Experimental studies focusing on microbial organisms that can form multicellular masses show that DPMs coupling can give rise to spatial structuring and cellular patterning. For instance, the cellular arrangement within fruiting bodies of the bacterium *Myxococcus xanthus* is partially determined by cell-to-cell communication via a contact signal that is also involved in cellular adhesion (C-signal) and that enables patterning mechanisms. This membrane-associated protein is localized at the cell poles; at low intensity it triggers the aggregation phase, while at high levels it triggers sporulation, leading to a concentric pattern that determines the final cellular arrangement in fruiting bodies (Julien et al., 2000). In the development of another model organism, *Dictyostelium discoideum*, prespores and prestalk cells are predetermined in a distinctive centripetal pattern with prespore cells confined to the center and the base and prestalk cells located in the periphery. This pattern is generated prior to the formation of the fruiting body by the enhanced differentiation in prestalk cells in the presence of the higher oxygen concentration at the periphery, and is reinforced by internal cellular coupling via ammonia and cAMP (Bonner et al., 1995, 1998; see review in Bonner, 2001).

In all of the above examples, initially indistinguishable cells are eventually specified as particular cell types or acquire a particular cellular behavior. The determination and organization of these cell types emerge as a result of the dynamic coupling of cells via direct or indirect cell-to-cell communication, suggesting that differentiation and patterning in early multicellular systems does not need to be traced back to differences among single cells, but that patterning may result from the collective dynamics of an initially homogeneous (or at least statistically homogeneous) group of coupled cells.

### 3.2. Multistable cellular networks and the molecules involved in the fundamental adhesion and communication DPMs are well-conserved and could had preceded the transitions to multicellularity

The key components of the adhesion and communication DPMs, critical for the emergence of multicellularity, appear to be present in a variety of multicellular plant and animal lineages and their closest unicellular relatives.

In the case of adhesion, the proteome of the unicellular choanoflagellates has many adhesion proteins, including cadherins, inmunoglobulins and alfa-integrins (Rokas, 2008b). In the choanoflagellates, some of these proteins seem to be used as a food-catching device. Similarly, some unicellular fungi have collagen (King et al., 2008; Rokas, 2008a) and the algae *Chlamydomonas reinhardtii* has a cell wall with the same components as the extracellular matrix of multicellular organisms, like the one observed in *Volvox* (Kirk, 2005). Similarly, communication molecules like Notch, Hedgehog, and MAPK, well known for their role in cellular communication, have been identified in unicellular organisms (Rokas, 2008a). Another example is the autoinducer molecules that serve as signals already present in bacterial populations (Shapiro, 1998). It is worth noting that in addition to the conservation of some critical molecules for adhesion and communication, simple cellular communication and adhesion could have emerged relatively simply via certain genetic or environmental changes leading to, for example, incomplete division and the formation of intercellular channels.

Finally, multistable gene expression and differential cellular states have been identified in many unicellular organisms (Sanford et al., 2002; Lopez et al., 2009; Maisonneuve et al., 2013). One example is in *C. reinhardtii*, where the presence of light activates the movement of the organisms toward the source of light, while the absence of light induces cell division, clearly showing two different cell states (Kaiser, 2001). In the same organism, nitrogen deficiency induces the differentiation of vegetative cells to pregametes, which are converted to gametes after a light impulse (Beck and Acker, 1992).

Multistable networks, cellular communication and adhesion molecules seem to be well-conserved among multicellular organisms and their closest unicellular relatives. Therefore, it is plausible to assume that these elements were already present before the transitions to multicellularity (Miller and Bassler, 2001; Abedin and King, 2008; Davidson and Surette, 2008; Newman and Bhat, 2009; Hernández-Hernández et al., 2012; Suga et al., 2013).

### 3.3. DPMs-based hypothesis for the development of patterns of differentiated cells in the transition to multicellularity

If, as argued above in Sections 3.1 and 3.2: (i) DPMs (specifically adhesion and communication DPMs) are capable of generating cell patterning among multistable cells and (ii) the molecules involved in the fundamental adhesion and communication DPMs are well-conserved among multicellular and their unicellular relatives (being likely then that these molecules were present before the transitions to multicellularity), then one can hypothesize that:

*In the transition to multicellularity, robust patterns of differentiated cells can result from the adhesion and communication DPMs that couple multistable cells and, thus, the origin of such patterns does not need to assume any pre-established or individual cell behaviors (cooperation or defection) nor fitness differences among cells*.

In the following section we present a dynamical model that incorporates the conditions (i) and (ii) reviewed above and illustrates the possible cell differentiation patterns generated by adhesion and communication DPMs in a mass of multistable cells. We also use this model to explore how changes in the parameters of the DPMs generate variation in the observed multicellular patterns. Importantly, the cells in the model are identical. Hence, they do not have any pre-established cell behavior or fitness difference. As other previous models (e.g., Furusawa and Kaneko, 2002; Zhu et al., 2010a), our model supports that patterns can result from the interactions of DPMs, but it aims to explore this process in the transition to multicellularity, so it only considers components associated to cell adhesion and communication, present in the unicellular and multistable virtual elements.

## 4. A dynamic and *in silico* exploration of our DPMs-based hypothesis

In the previous sections we provided empirical and theoretical evidence that supports the conditions (i) and (ii) underlying our hypothesis. The following model illustrates how the dynamical coupling of cells by DPMs can, in principle, give rise to complex and steady patterns, even from cells that are initially indistinguishable from each other.

### 4.1. Model description

**Figure 1:**
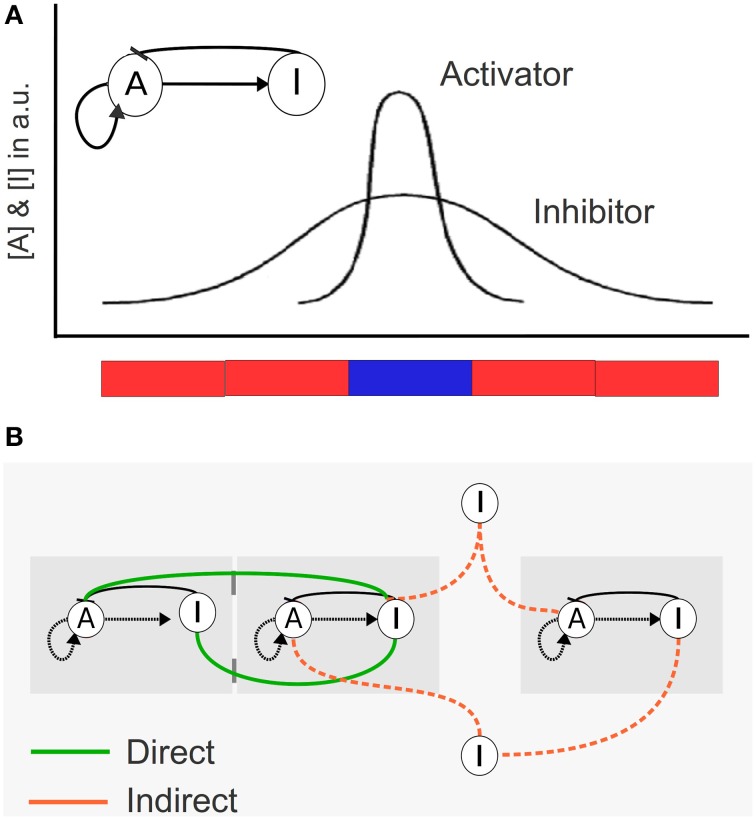
**Schematic description of the mathematical model for cellular coupling via different modes of communication. (A)** Internal network composed by an Activator (*A*) and an Inhibitor (*I*) in each cell. The *A*/I ratio determines the color of the cell (if *A*≥ I: blue cell, if *I* > A: red cell). In this figure we represent the concentration of *A* and *I* in arbitrary units (a.u.) of five cells in a filament, where each cell is blue or red depending on the *A*/I relation. **(B)** Different types of cellular communication. Cells can communicate in three ways: (i) direct communication mediated by the diffusion of a signal to adjacent contacting cells (green), (ii) indirect communication in which the signal can be diffused to and sensed from the medium (orange), and (iii) a mixed scenario in which both direct and indirect communication are allowed.

We model a population of identical cells, each with a simple intracellular regulatory network. The network has the structure of the so-called activator-inhibitor system (Figure 1) (Meinhardt and Gierer, 2000). This network has two nodes, *A* and *I*, that can correspond to metabolites, proteins or other molecules, and reaches two steady states. We define each steady state as different cell types, identified here as activated (*A* ≥ I; blue) and not activated (*I* >A; red). (For a detailed explanation see **Box 1**).

The virtual cells are set to interact under different scenarios of cell adhesiveness and communication. Cell adhesiveness could vary in strength (none, low, high) and as adhesiveness values increase, cell movement within the group decreases regardless of the specific nature of the molecules and processes involved. In the high adhesion scenario we fix the cells, avoiding membrane fluctuations. In the other scenarios, cell shape is variable over time (**Box 1**). The coupling of cells in a mass can be considered as a consequence of intercellular communication, which can be defined as direct or indirect. In direct intercellular communication, cells communicate only with adjacent cells they are in contact with, for example via membrane proteins or by intercellular channels. In indirect communication, cells secrete a signal that once in the medium can be detected by surrounding cells. In our model, communication is (i) direct, (ii) indirect or (iii) mixed, when both direct and indirect communication are present. Our model thus contains multistable cells and the key elements of a DPM: molecules present in single cells associated with adhesion and communication mobilizing a generic patterning mechanism (here, activator-inhibitor) as cells transition to the multicellular scale. We performed a set of simulations using CompuCell3D (CC3D), a software that allows modeling of multiscale biological systems using the Potts formalism (Swat et al., 2012).

### 4.2. Simulations and results

**Figure 2:**
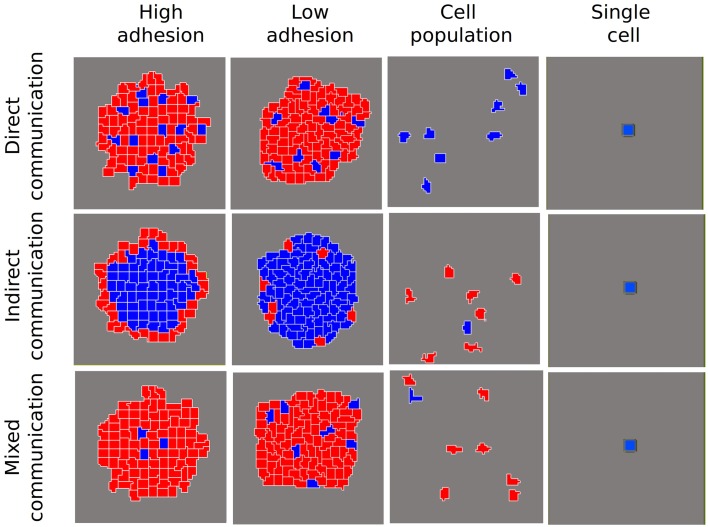
**Patterns of differentiated cells emerging in different scenarios of cell-to-cell communication and for different degrees of adhesion among cells**. We show a control scenario of individual cells where, independently of the communication type, cells always reached a steady state where *A* ≥ *I* (blue cells). Another control scenario shows the simulation of a population of cells without adhesion. In this case, when indirect communication is allowed, different cell states are reached (blue cells: *A* ≥ *I*; red cells: *I* > *A*), without any patterning. When cellular adhesion is introduced and is high, three different patterns of differentiated cells are attained. If adhesion is low (i.e., cellular movement allowed) these patterns are qualitatively similar, but are not stable.

We carried out simulations first for a single cell and then for a cell population. In a single cell, the network dynamic always leads to the blue cell type (*A* ≥ I), irrespective of the kind of communication being simulated (Figure 2). Similarly, in a cell population (non adhesive cells) without any kind of communication (*s* = 0, *d* = 0 in Equations 5 and 6 in Box 1), the network dynamic always reaches the blue cell attractor. Nevertheless, when indirect communication is allowed, the population can become heterogeneous (Figure 2). Then, when we include cell adhesion, diverse cellular arrangements with the coexistence of different cell types emerge. As suggested by Furusawa and Kaneko (2002) for a model of cellular masses, when there is both intercellular adhesion and communication, cell patterns with differential gene expression states arise. In our simulations, the nature of the particular arrangements depends on the kind of communication (*s* and *d* in Box 1).

Box 1 **GGH Simulation and Description of the Model**

**1. Virtual Cells**

**Figure 3:**
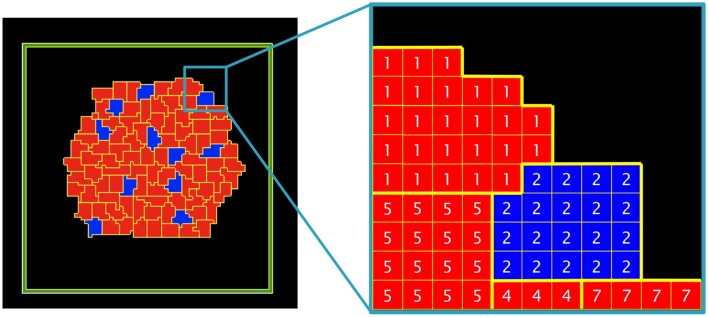
**Detail of the cells and the external medium in a bi-dimensional lattice**. Five different cells are depicted (τ = 1, 2, 4, 5, 7) belonging to two different cell types (σ = blue, red), a non-flux frontier (in green) and the external medium (in black).

In Glazier–Graner–Hogeweg (GGH) simulations, cells are defined as a set of pixels in an *ixj* lattice (Figure 3). Each cell has its own identifier (τ) and belongs to a particular cell type (σ). In our model, there are two cell types (blue or red) and an external medium. The cell type depends on the ratio of two internal molecules; activator *A* and inhibitor *I* (if *A* ≥ I: blue, if *I* > A: red).

**2. Effective Energy: Adhesion, Volume**

The core of GGH simulations is the *Effective Energy H_GGH_* which describes some cell behaviors and interactions. The objective of the GGH simulations is to find the configuration of cells and cells types with the minimum *H*_GGH_. As such, characteristics of cells are described by different instances of *H*:

(1) $$H_{GGH}=H_{boundary}+H_{constraints}$$

To begin with, we define a boundary energy *H_boundary_* to simulate cell adhesion between cells of different types:

(2) $$H_{boundary}=\sum_{\vec{i},\vec{j}\;neighbors}J\left(\left(\tau \left(\sigma (\vec{i})\right)\right)\left(\tau (\sigma (\vec{j}))\right)\right)\left(1-\delta \left(\sigma (\vec{i}),\sigma (\vec{j})\right)\right)$$

Where *J*((τ(σ($\vec{i}$))), (τ(σ($\vec{j}$))) is the boundary energy per unit area between two cells (σ_1_, σ_2_) of given types (τ(σ($\vec{i}$))),(τ(σ($\vec{j}$))) at a contact point. The term (1−δ(σ($\vec{i}$), σ($\vec{j}$))) avoids taking into account pixels belonging to the same cell. If *J* between cells is high compared to the contact between the cells and the medium, adhesion between both cells will be low and *viceversa*. In our model, we perform three different adhesion scenarios (high, low and no-adhesion).

Volume, surface and other characteristics are defined through an elastic form, where each of the variables tends toward an objective value. For example in the case of volume (*v*):

(3) $$H_{constraint=v}=\sum_{\sigma }\lambda {\left(v(\sigma )-v_{tg}(\sigma )\right)}^{2}$$

Where *v*(σ) is the actual volume of the cell and *v_tg_*(σ) is the target volume. λ is the spring constant which determines the constraint strength.

**3. Dynamics and shape**

The configuration of the lattice changes over time through random index-copying attempts between pixels (for example pixel $\vec{i}$ tries to copy its own identifier (τ) to $\vec{j}$) which are accepted or rejected according to the Boltzmann probability:

(4) $$P(\sigma (\vec{i})\to \sigma (\vec{j}))=\{1:\Delta H\leq 0,e^{-\Delta H/T}:\Delta H>0\}$$

Where *T* is the *membrane fluctuation* parameter. These dynamics enable a cell to move, grow and change shape during the simulation. Since cells' membranes are in constant fluctuation, their shape is only determined by the optimization of the target volume and target surface. In the high adhesion scenario, we established that cells do not move, so we fixed the cells in order to eliminate the unnecessary fluctuations. When a cell is fixed, there are no attempts for index copies (as we will see below). As a result, cells keep the same shape all over the simulation.

***4. Cells internal network and cell communication***.

Each of the cells σ in the simulation has an identical Activator-Inhibitor network (Figure 1), defined in CC3D through Ordinary Differential Equations (ODEs) and Partial Differential Equations (PDEs). This network has two nodes or genes, an Activator (*A*) and an Inhibitor (*I*). *A* promotes the production of itself and that of *I*. On the other hand, *I* inhibits the production of *A*. This is summarized by Equations (5) and (6) (without the communication terms).

We considered three types of communication: direct, indirect and mixed. This model assumed the existence of a signal (specifically, the signal was the node *I*) that moves between cells through diffusion without any receptors. All scenarios are included in Equation (6). When communication is direct, *I* can only move through cells that are in direct contact with each other (*s* is set to 0; and only the second term is considered in Equation 6). When communication is indirect, cells can only communicate with the outside medium (*d* is set to 0; and only the first term is considered in Equation 6). When communication is mixed, cells can communicate both with their direct neighbors and with the external medium (both terms are considered).

(5) $$\frac{dA_{\sigma }}{dt}=\frac{k_{1}A_{\sigma }^{2}}{I_{\sigma }}-k_{2}A_{\sigma }$$

(6) $$\frac{dI_{\sigma }}{dt}=k_{3}A_{\sigma }^{2}-k_{4}I_{\sigma }+\overset{\text{Indirect Comunication}}{\overset{︷}{s(I_{ext_{\sigma }}-I_{\sigma })}}+\overset{\text{Direct comunication}}{\overset{︷}{d(\sum I_{n}-\#(N_{\sigma })I_{\sigma }),n\in N_{\sigma }}}$$

Where *A*_σ_ and *I*_σ_ are the concentration of *A* and *I* in cell σ. *I*_ext_σ__ is the concentration of *I* outside the cell σ – in its boundary. *N*_σ_ is the set of neighbors of cell σ. *n* is a cell identifier that belongs to *N*_σ_. $\sum I_{n}$ is the sum of *I* in the neighbors of cell σ. #(*N*_σ_) is the number of neighbors of cell σ. *k*_1_, *k*_2_, *k*_3_, *k*_4_, *s* and *d* are parameters that could account for bistability. Neighbors are continually changing in time and are calculated in each step.

When *I* is present in the external medium, it only diffuses passively through the medium (Equation 7). In the boundary in contact with the cell σ, there is an exchange of *I* with the cell σ (Equation 8).

(7) $$\frac{\partial I_{ext}}{\partial t}=D\nabla^{2}I_{ext}-k_{5}I_{ext}$$

(8) $$\frac{\partial I_{ext_{\sigma }}}{\partial t}=D\nabla^{2}I_{ext_{\sigma }}-k_{5}I_{ext_{\sigma }}+s(I_{\sigma }-I_{ext_{\sigma }})$$

Where *I*_*ext*_σ__ is the concentration of *I* in the boundary with cell σ. *D* corresponds to a diffusion rate and *k*_5_ stands for the degradation rate of *I* in the medium.

Finally, it is important to emphasize that we employed these Equations (5) and (6) due to their bistability that could account for the different cell types.

***5. CC3D global dynamics***.

Swat et al. (2012) contains a useful and detailed explanation of the CC3D method. In brief, CC3D performs simulation in discrete time steps, the so-called MonteCarlo Steps (Swat et al., 2012). In each of the steps there are as many random index copy attempts (accepted or not according to the Boltzmann equation probability) as there are pixels in the lattice. Then the ODE and PDE are calculated through the Forward Euler Method and cell types are updated depending on their *A*/I ratio. The cycle then repeats, for approximately 30,000 steps.

As shown in the final states depicted in Figure 2, direct communication gives rise to a pattern of spaced blue cells surrounded by red cells. Indirect communication renders radial patterns in which the red cell type arises preferentially on the border of the aggregate, surrounding the blue cell type. Finally, mixed communication results in an interesting pattern of blue dots tending to locate in the center of the aggregate.

When adhesion decreases, there are differential effects on the final pattern, depending on the kind of communication. The decrease in cell-to-cell adhesion provokes an increment in cellular movement and, as a consequence, a continuous change in the relative position of cells in the aggregate. In the direct communication scenario, this continuous change does not affect the qualitative pattern obtained. Nevertheless, in the case of indirect and mixed communication scenarios, where the position in relation to the external medium is critical, the final pattern is highly modified by the continuous movement of cells in the aggregate. Finally, our simulations indicate that such arrangements are robust to changes in the aggregate size and transitory perturbations (data not shown).

In accordance with previous work (e.g., Salazar-Ciudad et al., 2001; Furusawa and Kaneko, 2002; Salazar-Ciudad, 2010; Zhu et al., 2010a), our results suggest that initially identical cells can give rise to a variety of patterns as a consequence of their coupling (in this case via adhesion and communication), which produces differences in local and internal properties. While DPMs render robust patterns, these arrangements can be modified by intracellular or environmental factors, providing a source of variation. In particular, our simulations suggest that the specific mode of communication has an effect on the emerging cellular arrangements and should be further investigated.

Interestingly, some of the patterns we observe (Figure 2) resemble the arrangement of some of the classical model systems in the study of multicellularity, even if the particular coupling mechanisms are different from the ones we modeled. A typical example of an emerging multicellular arrangement is that of spore cores surrounded by accompanying cells in *M. xanthus* (Sager and Kaiser, 1993; Julien et al., 2000; Holmes et al., 2010). This arrangement is similar to that generated in the mixed communication scenario, though our model used different specific coupling mechanisms than occur in *M. xanthus*. However, our simulations support the hypothesis that this particular arrangement could be, in principle, originated from similar cells coupled by different types of communication. Another interesting example comes from the predetermination of preespore and prestalk cells in *D. discoideum* before the formation of the fruiting body. This arrangement, where peripheral cells become prestalk cells and internal cells become prespore cells, is determined by the effect of oxygen in the medium, reinforced by internal loops of ammonia and cAMP (Bonner et al., 1995, 1998; Bonner, 2001). This emphasizes the role of the position of the cells with respect to the medium in their determination. The pattern is similar to our indirect communication scenario where the contact with the medium is decisive (Figure 2).

## 5. Impact of our hypothesis on the cooperation–defection framework

According to our hypothesis and simulations, the development of cell specialization and patterning in emerging multicellular organisms could be a mechanistic consequence of cell interactions and physicochemical properties. In all our modeling scenarios, stable and non-trivial patterns of cellular states emerged without assuming that individual cells had an intrinsic initial cooperative-defective strategy. Hence, the differentiation between cell types (for example, between somatic and germinal cells) and the spatial arrangement in emerging multicellular organisms using a DPMs approach do not need to be interpreted under a cooperation-defection framework. Consequently, we argue not only that the DPMs approach relaxes some of the cooperation and defection assumptions often invoked when explaining the development of cellular differentiation and patterning in emerging multicellular organisms, but even that some of these assumptions could be incompatible with the DPMs-based hypothesis.

To begin with, under the cooperation-defection framework, individual cell behaviors (cooperator or defector) are usually pre-established independently of their context (e.g., internal chemical networks or coupling mechanisms). Assigning a particular behavior and fitness to individual cells in an aggregate implies conceptualizing them as independent agents and averaging a non-additive property among single cells. However, once cell coupling mechanisms are considered, a cell's behavior, and thus its fitness, is dependent on the internal properties of the cell, the cellular context and the interactions with other cells. Since under this vision cellular states are not individually acquired features but inseparable parts of a whole, an important consequence is that under the DPMs framework, defector cells would not give rise to defector cell populations. Instead, the identity and behavior of the cells, as well as their relative proportions, in an integrated multicellular organism would depend again on the cell properties and the cellular context and interactions. In addition, the cooperation-defection framework invokes paired relationships between cells (Nowak, 2006; Michod, 2007), while the non-linear nature of the processes that characterize developing organisms makes it seem unlikely that collective or systemic features are the result of paired interactions (Axelrod, 2006; Jaeger and Reinitz, 2006).

Finally, we argue that the DPMs approach can help to avoid or relax assumptions such as the presence of initial strategies due to genetic differences or the capacity of cells to establish beneficial relationships with those cells that are more genetically similar. In principle, cell-to-cell coupling can arise and render patterns of cell differentiation in cells that are initially identical as in our simulations, or in cells that are heterogeneous. The fact that in both empirical (e.g., *Volvox* or filamentous cyanobacteria) and theoretical (e.g., our simulations) systems patterns are generated from initially homogeneous groups of cells suggests that initial genetic strategies are not necessary for pattern formation.

In our opinion, the consequences of our hypothesis on the cooperation–defection framework open other questions that could be explored in future research. Hence, in the last section, after a brief recapitulation of our ideas, we discuss some of these possible questions.

## 6. Discussion

The cooperation-defection and DPMs frameworks are the two main frameworks for explaining the origins of cell differentiation and patterning in the transition to multicellularity. While the cooperation-defection framework focuses mainly on evolutionary questions, the DPMs one is more interested in the developmental mechanisms that could be involved in cell differentiation and patterning (although it may also have evolutionary implications). In this work we postulate and test the hypothesis that cell differentiation and patterning in emerging multicellular organisms are the consequence of the coupling of multistable cells via communication and adhesion DPMs. These DPMs, along with intracellular networks, are fundamental ingredients for multicellularity to arise. Our hypothesis is supported by demonstrations of cell patterning as a result of adhesion and communication DPMs and their coupling with multistable cells, as well as on evidence suggesting that the coupling molecules and multistable behaviors pre-date multicellular organisms and could be coopted in the transitions to multicellularity. Once the cells are coupled, cell behaviors are dependent on the interactions between cells and hence, it is unnecessary to assign inherent behaviors or fitnesses to individual cells. We implemented a model considering the assumptions entailed by our hypothesis and further explored *in silico* its outcomes and some possible sources of phenotypic variation (communication types and adhesion strength). Our simulation results, along with the current available evidence, support our hypothesis and enable us to further discuss the possible implications of our proposal on the cooperation-defection framework.

Our simulations show that particular cellular arrangements could be originated from initially identical cells that can both directly and indirectly communicate, spontaneously reaching different identities in the aggregate (Figure 2). Moreover, if the external and internal conditions are maintained, this arrangement could be in principle re-created in a population of interacting cells with certain local and shared properties (e.g., adhesiveness and a type of response to communication). The variability in the cellular patterns can be generated by changes in particular DPMs (e.g., type of communication), which can in turn be due to changes in the single cells of a population, or to environmental changes that favor different types of coupling (Newman and Müller, 2000; Newman and Bhat, 2008, 2009). Further theoretical and empirical explorations might help to clarify how different types of communication and sources of variation affect the generated patterns of cell differentiation, as well as to identify robust cellular patterns and the cellular and environmental processes that are necessary and sufficient to reproduce them.

The DPMs framework emphasizes the role of cellular interactions and physicochemical processes between cells and the external environment in producing phenotypic variation in the first multicellular organisms (Newman et al., 2006). Indeed, Newman et al. (2006) do not deny the role of genetic changes in the organism variation, but they hypothesize that genes might act as regulators (rather than creators) of change, through the processes of stabilizing selection (Schmalhausen, 1949), canalization and genetic assimilation (Waddington, 1953; West-Eberhard, 2003). They explain that if individuals with certain patterns of cell types, developed through the action of DPMs, have some advantage (or no disadvantage) they will act as a template that permits genetic changes that further regulate and make those pattern more robust. For example, the internal genetic network could be modified and make the pattern more reliable and robust against internal mutations (Salazar-Ciudad, 2010). This would in principle stabilize the process of re-creation with DPMs through generations.

Considering fitness is certainly important for the evolution of multicellularity in populations, as natural selection will modify these populations selecting for the fittest individuals. Yet our proposal does not rely on fitness as the sole explanation for the development of phenotypic variation in emerging multicellular organisms. The evolutionary scenarios discussed above, as well as those considering competition, nutrient availability, populations of reproducing aggregates, etc., could be explored in future versions of this and other dynamical models and would shed light on the evolutionary implications of our proposal.

Another important issue that could be addressed in future modeling efforts refers to the role of stochastic fluctuations among genetically homogeneous cells. This has indeed been reported as a relevant factor in the determination of cell types in some multicellular organisms formed by aggregation (Nanjundiah and Sathe, 2013).

Our results imply that it is possible to reach and re-create cell differentiation and cell patterning, in a variety of spatial arrangements, without appealing to cooperative or defective behaviors. To our knowledge, this is the first time this proposal is discussed in the context of the transitions to multicellularity and contrasted with the cooperation-defection framework. Nevertheless, the role of conflict-mediating mechanisms in systems of coupled cells should still be carefully studied and discussed, for example in a set of independent, non-coupled individuals.

If there is some initial heterogeneity between largely independent cells due for example to mutations (Travisano and Velicer, 2004), cell behavior could be associated to such initial cell heterogeneity, and the cooperation-defection framework could apply. Nevertheless, this framework is better suited to explain the persistence of cooperative populations, in spite of the presence of cheaters (Foster et al., 2004; Travisano and Velicer, 2004; Santorelli et al., 2008) and not the mechanisms that lead, or have led, to the evolution of spatial cell differentiation. For example, mutant cheating strains have been identified in *M. xanthus* and in *D. discoideum*. When mixed with wild type strains, these cheating strains are overrepresented as spores and underrepresented in the stalk (Strassmann et al., 2000; Velicer et al., 2000). This imbalance might lead the population either to collapse (due to its inability to form fruiting bodies for dispersion) or to a complete switch from the wild type to the mutant strain where there would be, again, two types of cells: stalk and spore cells in normal proportions (e.g., facultative cheaters, Santorelli et al., 2008). However, in neither of both cases, the presence of cheaters affects nor explain the patterns of cell differentiation themselves.

The cooperation-defection framework has also been used to explain differences in the degree of complexity attained by different organisms and lineages. For instance, there are two ways by which groups of cells can arise, namely, aggregation of initially isolated cells (e.g., *M. xanthus* or *D. discoideum*) and incomplete division (e.g., animals and plants, which develop from a single cell). Since the multicellular organisms that are often identified as the most complex in terms of size, number of cell-types and body plans are generated by incomplete division, it has been speculated that the minimization of genetic conflict caused by genetic homogeneity allows them to generate more cell types and spatial structures (Grosberg and Strathmann, 2007). Aggregates formed from cells that can descend from different lineages are thought to have more genetic conflict and thus reach less complexity. There are complementary ways to tackle this problem. Some sources suggest that genetic homogeneity is not a necessary condition for cell differentiation (for example in the case of *D. discoideum*, Bonner, 2001) nor a sufficient condition (*Eudorina elegans*, a 32 cell homogeneous aggregate, does not have any spatial cell differentiation, Kirk, 2005). From the DPMs framework we could speculate than some differences between aggregates and organisms arising from incomplete division might be due to the dynamic differences between clusters that result from cells that become coupled in a single and relatively fast event (aggregation) vs. clusters resulting from cells that are gradually incorporated and coupled into the cluster (incomplete division).

Similarly, the relationship between size of the aggregate and cell differentiation has been well documented (Bonner, 2004). In the context of the cooperation-defection framework, it has been suggested that once a mass of undifferentiated cells reaches a threshold size, division of labor becomes beneficial for the group even if it implies that some of the cell types will have relatively low fitness, leading to or maintaining cell differentiation (Michod, 2007). However, another explanation based on the dynamical properties of coupled cells is also possible; Kaneko has suggested that in larger aggregates of coupled cells, more microenvironments of nutrient concentration or signals can emerge from cell-to-cell and cell-medium interactions, which in turn bias the cellular fates and yield more cell types (Furusawa and Kaneko, 2002).

Finally, it is worth noting that our work and discussion focuses on the process of cellular differentiation and patterning in emerging multicellular organisms and that any extrapolation to other biological or social scales are beyond the scope of our model (though it would be interesting to address how the coupling of different dynamic mechanisms could change our understanding of collective organization at other scales).

We have pursued a modeling approach based on the DPM framework to address one of the questions we consider central in evolutionary developmental biology: the origin of cell differentiation and patterning in the transition to multicellularity. This approach relies on different assumptions than the cooperation-defection framework on the problem of cell differentiation and provides new working hypotheses, complemented with dynamical mathematical modeling. This approach is specially interesting for the transition to multicellularity, as Bonner (2001) put it: *“In trying to reconstruct the beginning, our most effective tool is mathematical modeling, which allows us to ask: What is the minimum signaling needed to produce a pattern?”* We believe that it would be interesting to evaluate these ideas with oriented experiments in order to validate the possible predictions. In fact, we consider that joint theoretical and experimental approaches (e.g., experimental evolution) will be key to uncovering some fundamental principles behind the development and evolution of multicellularity.

### Conflict of interest statement

The authors declare that the research was conducted in the absence of any commercial or financial relationships that could be construed as a potential conflict of interest.
